# Flexible data handling for routine quantitative analyses employing a gas chromatograph-mass spectrometer under computer control

**DOI:** 10.1155/S1463924681000242

**Published:** 1981

**Authors:** Jan Vink, Paul C. J. M. Koppens, Frank A. van Harmelen, Wim E. van Voorthuijsen

**Affiliations:** 1 Organon International B. V. Drug Metabolism R&D Labs P.O. Box 20 Oss 5340 BH The Netherlands; 2 AKZO Corporate Research Applied Physics P. 0. Box 60 Arnhem 6800 AB The Netherlands


### Flexible data handling for routine quantitative analyses employing a gas chromatograph-mass spectrometer under computer control

**Jan Vink* and Paul C. J. M. Koppens**
*Organon International B. V., Drug Metabolism R&D Labs, P.O. Box 20, 5340 BH Oss, The Netherlands.*

**Frank A. van Harmelen and Wim E. van Voorthuijsen**
*AKZO Corporate Research, Applied Physics, P.O. Box 60, 6800 AB Arnhem, The Netherlands.*

### Introduction

Gas chromatography – mass spectrometry (GC-MS) has found many applications for the quantitative determination of compounds of pharmacological interest in biological fluids [1 - 4]. With quantitative mass spectral methods, also referred to as mass fragmentography, selected ion monitoring, multiple ion detection or multiple peak scanning [5], a high degree of sensitivity and specificity can be obtained to enable quantitative analyses at low levels without interference from natural constituents of biological origin. Modern GC-MS systems can be equipped with hardware devices and software packages for control of the mass spectrometer by a datasystem to facilitate the quantitative analyses. However, processing of large series of biological samples is still hindered because the standard software is of little further assistance to the user once raw mass spectral data for quantitative analyses are obtained and converted into gas chromatographic retention times, peak heights and areas at the masses monitored. The user has to struggle through the cumbersome selection of the gathered data, calculate a calibration function and check the obtained calibration before being able to obtain quantitative measurements. It is clear that this approach is not well suited for performing quantitative GC-MS analyses in a routine way. In this paper the authors describe the extension of the Varian SpectroSystem 100MS software for routine quantitative analysis of drugs in plasma using a GC-MS system operated under control of the Varian SpectroSystem 100MS.

### Hardware, software and methods

### Hardware

The Varian SpectroSystem 100MS consisted of Varian 620/L-100 computer with 16K of 16 bit words core memory. The mass spectrometer operated under computer control via a 14 bit D/A converter. Analogue signals from the mass spectrometer were transferred to the computer by a 12 bit A/D converter. The SpectroSystem 100MS was equipped with a Data Recording Diablo Dual Disc Unit Model 4043, a Tektronix 4010 terminal connected to a Tektronix 4631 hardcopy unit and a Pertec magnetic tape unit Model 6860-9.

Because the DISKOS software of the SpectroSystem 100MS was originally developed for a Tektronix 611 storage scope and Teletype as peripherals, the Tektronix 4010 terminal was extended with an external analogue input board (Tektronix part CM 018-0108-00), while the graphic mode was activated manually via-keyboard.

**To whom correspondence should be addressed.*

### Biological samples, assay method and equipment

To illustrate the capability of the GC-MS system operating under computer control for the determination of drugs in biological samples, plasma samples were analysed routinely. These samples originated from an *in vivo* experiment to determine the bioavailability of the antidepressant drug mianserin in the Beagle dog [6]. Oral administration of non-labelled mianserin and intravenous administration of tetradeuterium-labelled mianserin was applied simultaneously. Mianserin and tetradeuterated mianserin were both quantitated by GC-MS using dideuterium-labelled mianserin as an internal standard according to the assay method described by Vink and Van Hal [6]. The GC-MS system consisted of a Varian Aerograph Model 2740 gas chromatograph coupled to a Varian CH-7 mass spectrometer using a dual stage Watson-Biemann separator. The gas chromatograph was equipped with a 2 m, 2 mm I.D. glass column packed with 1% JXR on Gas Chrom Q, 80-100 mesh. The temperatures of column, injector and detector were 210, 270 and 270°C, respectively. Helium was

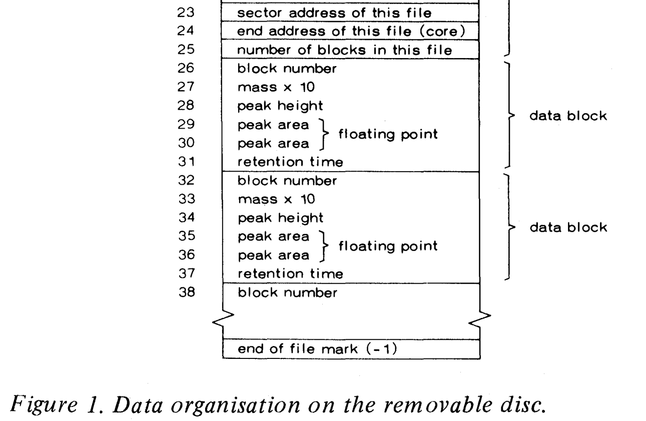

*Figure 1. Data organisation on the removable disc.*

used as carrier gas at a flow rate of approximately 30 ml/min. The mass spectrometric conditions were: electron energy 70 eV, ionising current 300 $\mu$A, electron multiplier voltage 2 kV, temperatures of ion source, separator and interface 135, 250 and 250°C, respectively. The mass spectrometer was focussed on mass 243 of the calibration standard perfluorokerosene applying a maximum accelerating voltage of 3 kV. Alternate switching of the accelerating voltage supplied by the high voltage unit BHH of the mass spectrometer under computer control enabled monitoring of the masses of the molecular ions of mianserin, dideuterated mianserin and tetradeuterated mianserin at mass 264, 266 and 268, respectively.

### DISKOS software

The standard Varian DISKOS software of the SpectroSystem 100MS was applied using the modules INIEM for system initialisation, ESET for defining the masses to be monitored, ECO for data collection and storage on the removable disc (raw "MI"-data), AUTI for integration of the intensity versus time data at each mass monitored, and WRTA for creation of a file consisting of integrated data (the "TA"-table) on the removable disc. Sense switch 2 of the console of the Varian 620/L-100 computer was activated to enable automatic focussing. The DISKOS modules INIEM, ESET, ECO, AUTI and WRTA were used in a sequence in order to create a file of integrated data (the "TA"-table) for a series of quantitative analyses without intervention of the operator.

### Data organisation on disc

In Figure 1, the data organisation on the removable disc of the Diablo Disc Unit is shown. The disc contains raw data written during data acquisition ("MI"-data) and integrated data calculated from "MI"-files by integration using the module AUTI to yield peak heights, peak areas and retention times of the GC-peaks at the masses monitored. A file of integrated data, the "TA"-table, consists of a header of 26 computer words of 16 bits and a number of data blocks. Each block contains an identification number, mass (multiplied by 10), peak height, peak area and retention time. After the last block the end of file mark -1 is written.

To provide an adequate check on the reliability of the results of analysis for the determination of nanogram amounts of a drug in biological samples, samples containing unknown concentrations of the drug, samples for establishment of the calibration curve, samples free of drug (blank), and blank samples spiked with a known amount of drug are analysed randomly on each day of measurement. Consequently, the "TA"-table of such a series of analyses contains the integrated data to be selected for calculation of the calibration curve and, by applying the calibration curve to data from samples, the calculation of the drug levels. In this way, the biological samples free of drug (blank) and blank samples spiked with a known amount of drug serve as controls on the overall analytical procedure. It is obvious that data selection and subsequent calculations can be carried out conveniently by the computer. With the standard DISKOS software of the SpectroSystem 100MS, the user has no access to the files via a simple computer language. Therefore, a new subroutine DISC was written to gain access to the "TA"-table, or a part of it. BASIC was selected as the computer language to obtain a flexible system.

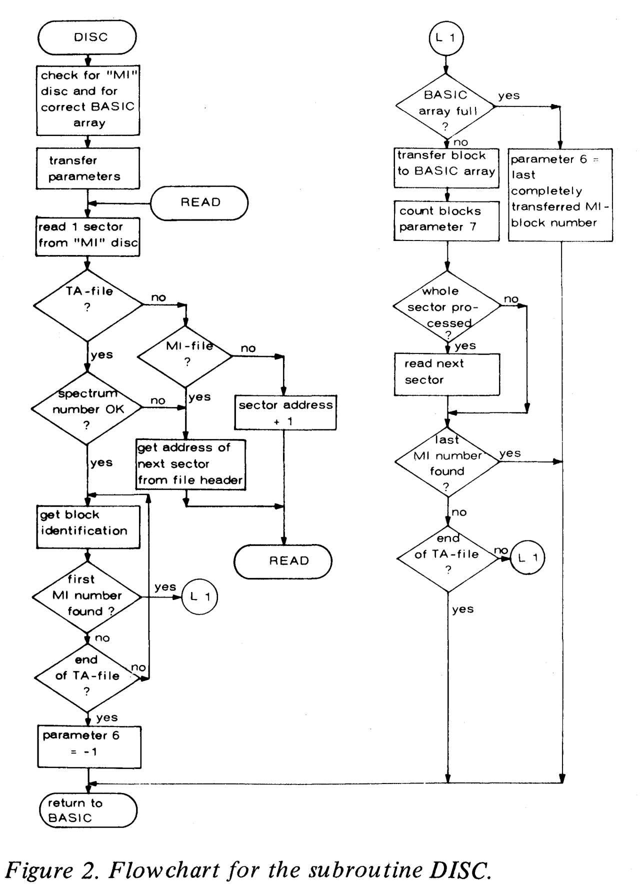

*Figure 2. Flowchart for the subroutine DISC.*

### Access to integrated data

A subroutine DISC was developed to read a specific TA-table (or part of it) from the removable disc, and to store these data in a BASIC array. A call to the subroutine DISC may be used as statement in a BASIC program, e.g. xxxx CALL DISC, $P_1$, $P_2$, $P_3$, $P_4$, $P_5$, $P_6$, $P_7$ where xxxx is the line number and $P_1$ ... $P_4$ are BASIC variables to be assigned prior to the subroutine call. $P_1$, $P_2$ and $P_3$ are the identification numbers of respectively the "TA" - table, the first data block from the "TA" - table which has to be transferred, and the last data block to transfer. $P_4$ is the name of the BASIC array. The parameters $P_5$ ... $P_7$ obtain their value in the subroutine. These parameters may be used in the BASIC program after the subroutine call has been executed. $P_5$ and $P_6$ indicate occurring disc and transfer errors respectively, $P_7$ yields the total number of blocks transferred. The flow chart for the subroutine DISC is shown in Figure 2.

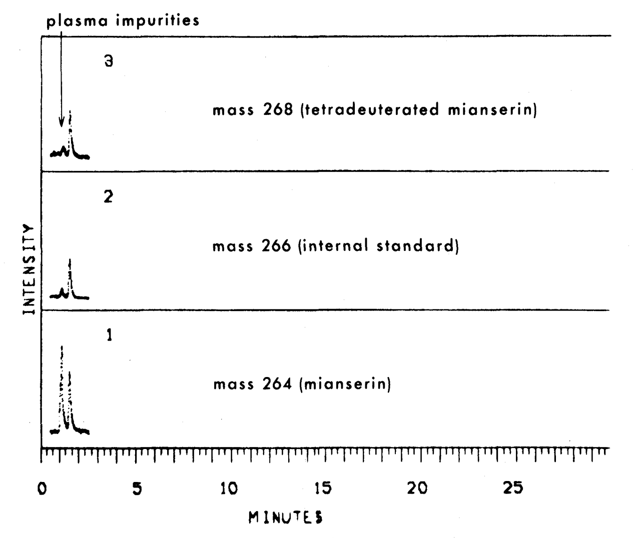

*Figure 3. Real time plot obtained after injection of a dog plasma extract calculated to contain 23,9 ng of mianserin and 20,2 ng of tetradeuterated-mianserin per ml plasma. To 1,5 ml plasma, 20 ng of dideuterated mianserin was added as internal standard.*

### Creation and updating of a library of BASIC programs on magnetic tapes

A subroutine MAGT was written to save BASIC programs, and to load these from magnetic tape. After the command CALL MAGT has been used on the Tektronix 4010 terminal, subcommands can be given to display a list of all BASIC programs stored, to save a new BASIC program under a particular name, to load a specific program from tape, to delete a program, to replace an old program by the one which is in core memory, or to return to the BASIC interpreter.

### Extension of BASIC with a graphic package

BASIC was extended with a graphic software package to display on the screen of the Tektronix 4010 terminal the calibration curve together with individual data points and alphanumeric text. This package enabled the operator to have a visual check on the calculated calibration curve in relation to the data used for calibration, with the possibility of reconsidering the data for calibration or to skip drop-outs.

### Data handling

During data acquisition and data storage, a real time plot is displayed on the screen of the Tektronix 4010. The real time plot displays the intensities at the masses monitored (maximum of 8 masses) and the total ion current (optional) versus time. An example is shown in Figure 3 where three masses are monitored of a dog plasma sample containing mianserin, tetradeuterated mianserin and the internal standard. After repeated data collection and conversion of the raw "MI"-data into integrated data which are stored as a "TA"-table on the removable disc, the BASIC program with the subroutine DISC incorporated is loaded from magnetic tape. A data block of the "TA"-table contains information on a GC-peak monitored at a specific mass and eluted at a particular retention time (Figure 1). In a dialogue with the computer, the user defines parameters for job identification and activates the transfer of the "TA"-table (or part of it) to core memory. In Figure 4 an example of the dialogue is illustrated. After this dialogue, the computer asks the user to define which of the selected data blocks contain data to be used for calibration. This is done by determining which of these data blocks correspond to a standard. This is accompanied by a simple yes or no questionnaire. After the data blocks used for calibration have been chosen this way, the computer lists these blocks and requests the user to define for each data block the x-axis position of the calibration function, e.g. concentration ratios of drug and internal standard. The corresponding peak height or area ratios, the y-values, are calculated and by non-linear least square fit the calibration function is calculated. The calibration function, the calibration curve and the individual data points are displayed on the Tektronix 4010 screen (Figure 5) giving the user the opportunity to judge visually the calibration and decide whether or not to go further or to

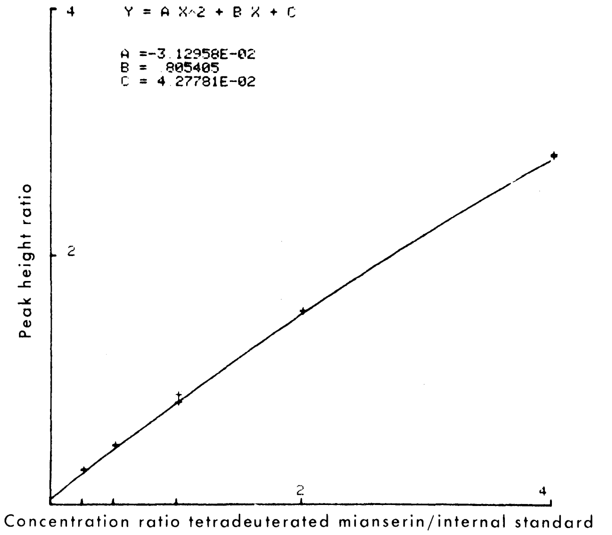

*Figure 5. Calibration curve for tetradeuterated mianserin using dideuterated mianserin as internal standard.*

```
RESULTS OF COMPUTER CONTROLLED GC-MS ANALYSES

DATE                          791206

EXPERIMENT NO.                DM 741

M/Z :DRUG - INT.STD.          268  - 266

RETENTION TIME :

   DRUG                       95   SEC

   INT.STD.                   95   SEC.

      TOLERANCE               10   SEC.

TA - TABEL                    10300

--------------------------------------

CALIBRATION CURVE CALCULATED BY NON LINEAR LEAST SQUARE FIT

BY MEASUREMENT OF PEAK HEIGHT

Y =-3.12958E-02   X^2  +  .805405    X  + 4.27781E-02

IDENTIFIER     CONC.RATIO     SIGN.RATIO

(MI-BLOCK)

 10301          2              1.52732

 10302          4             .2.75691

 10303          1              .790665

 10304          4              2.77156

 10309          .5             .434792

 10313          .25            .234629

 10319          .25            .243223

 10320          .5             .442193

 10321          1              .855161

 10322          2              1.52212

--------------------------------------

SAMPLES :

-------

IDENTIFIER     SIGN.RATIO     NG INT.STD     ML SAMPLE     DRUG NG/ML

 10305          .832681        40             .5            81.7031

 10306          8.02906        10             2             OUT OF RANGE

 10307          3.69529        10             1.5           OUT OF RANGE

 10308          1.19127        20             1.5           20.2026

 10310          .776159        20             2             9.45301

 10311          4.42986        40             .5            OUT OF RANGE

 10312          .888349        10             2             5.48303

 10314          .689095        10             2             4.14597

 10315          1.3457         10             2             8.67327

 10317          0              0              2             OUT OF RANGE

 10318          .358209        10             2             1.98898

---------------------------------------------------------------------
READY
```

*Figure 6. An example of a final report of quantitative GC-MS analyses.*

```
YES = 1  ,  NO = 0

DATE                                  ? 791206

EXPERIMENT NO                         ? 741

MASS (DRUG)                           ? 268

MASS (INTERNAL STANDARD)              ? 266

RETENTION TIME DRUG (MIN/SEC)         ? 1,35

INTERNAL STANDARD                     ? 1,35

RT-TOLERANCE                          ? 10

NUMBER OF STANDARDS                   ? 10

NUMBER OF SAMPLES                     ? 12

CALCULATION USING

PEAK HEIGHT                           ? 1

TA-TABLE (NO)                         ? 10300

FIRST DATA BLOCK (NO)                 ? 10301

LAST DATA BLOCK (NO)                  ? 10322

```

- DATE, EXPERIMENT NO } user's code

- MASS (DRUG), MASS (INTERNAL STANDARD) } masses monitored

- RETENTION TIME DRUG, INTERNAL STANDARD, RT-TOLERANCE } retention time and tolerance for drug and internal standard

- NUMBER OF STANDARDS, NUMBER OF SAMPLES } number of standards for calibration and samples in this series

- PEAK HEIGHT → selection of peak heights or alternatively peak areas

- TA-TABLE → indentification of TA-table

- FIRST DATA BLOCK, LAST DATA BLOCK } part of the TA-table used

*Figure 4. Job identification and data transfer.*

reprocess the data and skip drop-outs. A non-linear least square fit was required to calculate the calibration function. With quantitative GC-MS methods curved calibration lines not passing through the origin are often encountered [7, 8]. If the calibration is considered to be correct, the computer lists the remaining data blocks while the user has to supply information on the amount of internal standard added and the volume of plasma sample initially processed. After the sample identification, the system presents the final report, a typical example of which is shown in Figure 6.

### Conclusions

A flexible system for routine quantitative GC-MS analyses was developed using the Varian SpectroSystem 100MS. The software was extended with user-oriented BASIC programs. With the BASIC programs loaded from magnetic tape, access to integrated data stored on disc was obtained. The system provides an interrogation dialogue for the user to set criteria for data selection, to calculate a calibration function and to calculate the final result of analysis. The system fulfilled the demands on quality control on the results of analyses for the quantitative determination of low drug levels in biological samples.

*Note added in proof*

*The GC-MS system as described in this paper was recently extended with a Hewlett-Packard 7670 A automatic sampler for injection of samples into the GC column. The autosampler was activated by a homemade acoustic pulse counter. The acoustic signals were generated by the Tektronix 4010 terminal under control of the Varian DISKOS software using the module BELL.*

ACKNOWLEDGEMENTS

The authors wish to thank Mr. J. Eberhard (Ingenieursbüro für Datentechnik Dr. Ing. H. Seufert, Karlsruhe, G. F. R.) and Mr. U. Markwardt and Mr. M. Schmädecke (Varian MAT GmbH, Bremen, G. F. R.) for their information on the organisation of the DISKOS software.

REFERENCES

[1] De Leenheer, A. P. and Roncucci, R. R., (1977), "Quantitative Mass Spectrometry in Life Sciences", Elsevier, Amsterdam.

[2] De Leenheer, A.P., Roncucci, R. R. and Van Peteghem, C., (1978), "Quantitative Mass Spectrometry in Life Sciences II", Elsevier, Amsterdam.

[3] Millard, B. J., (1978), "Quantitative Mass Spectrometry", Heyden, London.

[4] Carrington, R. and Frigerio, A., (1977), *Drug Metabolism Reviews*, 6, 243.

[5] Beynon, J. H., (1978), *Pure and Applied Chemistry*, **50**, 65.

[6] Vink, J. and Van Hal, H. J. M., in "Quantitative Mass Spectrometry in Life Sciences II", Ed. De Leenheer, A. P., Roncucci, R. R. and Van Peteghem, C., 1978, Elsevier, Amsterdam, pp. 367–378.

[7] Picart, D., Jacolot, F., Berthou, F. and Floch, H. H., *ibid*, pp. 105–118.

[8] Pickup, J. F. and McPherson, K., (1976), *Analytical Chemistry*, **48**, 1885.

### An evaluation of the Gemsaec 3E centrifugal analyser

**Peter S. West, Jennifer A. Nisbet and John A. Owen**

*Department of Chemical Pathology, St. George's Hospital, London SW17, UK.*

### Introduction

Centrifugal analysers have been progressively developed since they were first introduced. The Gemsaec 3E centrifugal analyser (Electro-Nucleonics International Ltd., Breda, Holland) has a number of innovations. In place of filters, a grating monochromator provides monochromatic light over a wide range and a solid state photodiode allows measurement of light absorption with low electronic noise. There is improved temperature control within the rotor and the rotor wash cycle uses water only, which avoids the toxic and fire hazard of methanol used in other models. Finally, it has its own computer with a floppy disc mass storage providing a wide range of programs including a statistical package and provision for storage and recall of a large amount of analytical data.

The analytical performance of the Gemsaec 3E centrifugal analyser has been assessed and the results are presented here together with comparative published data relating to similar instruments.

### Description of the instrument

The Gemsaec 3E centrifugal analyser comprises five modules - rotoloaders, analyser, control module, computer and keyboard printer. It uses 16-place re-usable transfer discs.

The rotoloader comprises two automatic pipetting units (MicroMedic Systems, Inc. Horsham, U.S.A.) one for dispensing reagent (200–600μl) and the other for diluting sample (3–50μl) with diluent (20–200μl). Loading a transfer disc takes about 3 minutes.

The analyser has an optical system consisting of a diffraction grating monochromator with a wavelength range from 335 to 785 nm and a band width of 5 nm. The temperature of the rotor is adjustable between 20 and 40°C.

The control module allows selection of the various test parameters including temperature, time of first absorbance reading and intervals between readings. It contains an oscilloscope display which allows visual monitoring of reactions in progress.

The computer (LS11, Digital Equipment Corporation London) has 40K bytes of memory and a twin disc drive unit as mass store. The keyboard printer (LA36 Digital Equipment Corporation London) has a rating of 30 char/s and is used to operate the system and to display and manipulate results.

### Evaluation procedure

### Analytical methods

The tests used in evaluating the instrument were selected so as to involve a number of different analytical principles, viz.

